# Supplementary material for: High Frequency Protein-Rich Meal Service to Promote Protein Distribution to Stimulate Muscle Function in Preoperative Patients
Source: Nutrients. 2021 Apr 8;13(4):1232. doi: 10.3390/nu13041232 (PMC8068324; doi:10.3390/nu13041232)
Supplement: Supplementary file 1 [file nutrients-13-01232-s001.pdf]

Supplementary table S1. Example of a day menu of the intervention.

**Meal occasion**	**Meal**	**Energy (kcal)**	**Protein (g)**
Morning snack	Coconut-mango shake	335	9.7
Lunch	Tuna salad<br>Provencal tomato soup	246<br>81	15.7<br>8.2
Afternoon snack	Burger with brie and fig chutney	248	7.2
Dinner	Tagliatelle with chicken and pesto	529	23.1
Dessert	Peach curd with granola	158	7.7
